# The Role of English as a Foreign Language Teachers’ and Learners’ Emotions and Language Achievement and Success

**DOI:** 10.3389/fpsyg.2021.756853

**Published:** 2021-10-06

**Authors:** Qian Huang

**Affiliations:** Zhijiang College, Zhejiang University of Technology, Shaoxing, China

**Keywords:** EFL students’ emotions, EFL teachers’ emotions, language achievement, language success, syllabus designers

## Abstract

It is preserved that one of the noteworthy influential subjects of success and achievement is emotions, and enhancing emotions is dominant in promoting the language learning of students in the classroom. Although emotions are an integral part of the practices of both educators and students, their function has been sidelined due to the emphasis on intellectual instead of emotional scopes of foreign language learning. Therefore, the present theoretical review tries to refocus on the role of emotions of teachers and learners and their effects on language success and achievement. Successively, the effectiveness of verdicts for educators, students, syllabus designers, and future researchers are deliberated.

## Introduction

Emotions such as pleasure, anger, and sadness are innate in human being, which means it is regular for psychological and social actions of people to be influenced by emotions, and education is no exception (Ge et al., 2019). Emotions are at the center of language education, and within previous decades, there is an emergent attentiveness in emotion inquiries in language teaching (Dewaele et al., 2019; Dewaele and Li, 2020). Emotions can affect learning on the whole (Pekrun et al., 2002), and language learning particularly (MacIntyre and Gregersen, 2012). Although emotions are particular, multifarious, and challenging to understand, they are important in boosting influential teaching and learning (Shao et al., 2019). The position of emotions in language education has been significantly renowned by scholars (Saito et al., 2018; Bigelow, 2019), but it has relatively received little consideration in language learning (MacIntyre, 2002; Dewaele, 2015) due to the fact that a growing concentration on emotions in teaching has lately appeared in the research literature, considering this investigation domain from different perceptions or methods that have generated supportive perceptions into the active, complex, and sophisticated nature of human emotions (Agudo, 2018). Moreover, another considerable justification of the ignorance of emotions is that they are innately very personal and irrational, and accordingly, they are neither simply visible nor assessable (Ross, 2015). While several scholars accredited the significant function of emotion in the language process (e.g., Dewaele, 2005; Swain, 2013), investigations of emotion and language learning are dropping back the swiftly developing arena of emotion in psychology and learning (Goetz et al., 2010; Pekrun et al., 2019). Concerning the issue, Benesch (2012) pinpointed that emotions have been undoubtedly disregarded in the literature because they have been hard to capture.

It is just about the previous four decades that emotions have concentrated principally on negative emotions such as anxiety (Daubney et al., 2017) and boredom in language learning (Pawlak et al., 2020; Derakhshan et al., 2021; Zawodniak et al., 2021), and it is approved that students experience a massive collection of different emotions all through their learning development (Mercer and Kostoulas, 2018; Li, 2020; Zhang and Tsung, 2021). Emotions are not only a cogent activity but also a societal one that occurs in individuals meeting in a societal context, in which feelings affect the educational experience of teachers and their reaction to the involvement of education and learning (Dörnyei, 2005). Applied linguists may have undervalued the applicability of feelings lately, owing to the power of intellectual and the untruthful belief that reviewing emotion is someway unsystematic (Sharwood Smith, 2017).

Undoubtedly, the condition has reformed with the arrival of positive psychology (PP) as researchers stimulated by the PP movement have stated that not all types of emotions are negative, and they have attempted to boost eudemonic well-being (Ergün and Dewaele, 2021), so the consideration of scholars in the language education, instructors and teachers have been moved into positive emotions (MacIntyre and Mercer, 2014; MacIntyre et al., 2016). The launch of PP in language education has developed indulgent of the array of emotions, educators and students confronted and predominantly the function that positive emotions can perform in helping education (Dewaele and Alfawzan, 2018; Dewaele and Li, 2020; Wang et al., 2021), subsequently, emotions have a central role in education progression and success (Derakhshan et al., 2020; Li, 2020).

Both students and teachers similarly encounter various kinds of emotions alternating from constructive to destructive ones (King and Chen, 2019). In fact, Fried et al. (2015) declared that studies in the emotion realm have frequently concentrated on the emotions of students on the one hand. Emotions inspire the awareness of learners and activate the learning route that governs what is achieved and what is recalled. Several studies in different realms such as neuroscience, teaching, and psychology have demonstrated that emotions are dominant in education (Seli et al., 2016; Tyng et al., 2017). On the other hand, above and beyond the prominence of emotions in the learning, it is viewed as the central element of operational education (Hosotani and Imai-Matsumura, 2011) and an essential element of teaching quality (Day and Qing, 2009; Hosotani and Imai-Matsumura, 2011), and a large body of investigations in recent times have focused on the emotions of teachers, as well (Keller et al., 2014; Taxer and Frenzel, 2015).

Emotions, for students, have been defined as the dynamic powers of inspiration and enthusiasm in education (Dörnyei, 2005). White (2018) pinpointed that positive emotion in this field functions to boost the capability of being conscious of and notify things in the situation and increase mindfulness of language materials. Other scholars (e.g., Fredrickson and Losada, 2005; Dewaele et al., 2017) maintained that progressive emotions inspire interest, willingness to communicate, and support self-directed learning educational achievement, while lack of success may provoke lots of diverse emotions in learners, which consequently may influence their presentation and manage their forthcoming guidelines (Pekrun and Perry, 2014).

In addition, teachers play a significant role in EFL classrooms in providing materials to learners, observing their progress, organizing interaction among learners, providing feedback, and evaluating their success (Derakhshan et al., 2019; Pishghadam et al., 2021a). However, the place of teacher in the language process is more than that as they are responsible for handling the emotive sense of the classroom, building a constructive setting in the language learning classes, making social rapports among classmates, and preferably, instruction with pleasure, and confidence (Dewaele, 2018). Regardless of the anger and apprehension that teachers occasionally had in the classroom, for most individuals, teaching is regarded as a foundation of positive emotions, and these support their attentiveness for teaching within the classroom (Richards, 2020). Indeed, inquiries have indicated that while in the classroom, teachers undergo different emotions, for example, pleasure, burnout, superiority, anger, frustration, and apprehension (Chang, 2009; Frenzel et al., 2009; Sutton and Harper, 2009; Beilock et al., 2010; Fathi et al., 2021). The emotions of educators can form their perception, enthusiasm, and interactions with learners (Sutton and Wheatley, 2003); influence teaching efficacy, well-being, and work engagement of teachers regarding intellectual and motivational encouragement, classroom supervision, and support, that result in the success and accomplishment of learners (Frenzel et al., 2009; Greenier et al., 2021). Hagenauer and Volet (2014), and it plays a vital role in shaping the sense of proficient characteristics, assurance, success, and well-being of educators (Day and Qing, 2009; Li et al., 2019). Additionally, there is a significant correlation between the emotions of educators and learners as learners are regularly conscious and inclined by the emotions of instructors (Postareff and Lindblom-Ylänne, 2011).

The foremost roles of both positive and negative kinds of emotions that exist in language education have been discriminated as “broadening” and “narrowing” (MacIntyre and Gregersen, 2012; Dewaele and MacIntyre, 2014), academically based on the “Broaden-and-Build Theory” (Fredrickson, 2003). In Fredrickson’s theory, she discriminates the roles of both types of emotions, asserting that the positive types can expand temporary thought-action collections of societies and shape their permanent individual foundations, fluctuating from behavioral and academic bases to societal and emotional properties, while negative ones are reversed, triggering simple persistence manners. Likewise, the other theory in this regard is the “Control-Value Theory” defining that human affection is utilized to differentiate kinds of emotions, that is to say, valence (pleasant vs. unpleasant) and activation (stimulating *vs.* disengaging). The control value theory perceives emotions as ranges of interconnected emotional developments, by which emotional, intellectual, motivational, and physical mechanisms are at prominence. This theory arranges for an integrative method for scrutinizing several types of emotions experienced in achievement settings, containing academic situations along with achievement circumstances in other life realms (Ismail, 2015).

Using these proportions concentrates four extensive sets of emotions: positive stimulating (e.g., satisfaction, confidence), positive motivating (e.g., enjoyment, comfort), negative motivating (e.g., nervousness, embarrassment), and destructive aspects (e.g., tediousness, boredom) (Pekrun et al., 2017). The theory suggests that these emotions impact intellectual bases, enthusiasm to study, and use of learning approaches of learners, and techniques and consequently affecting their success (Pekrun and Linnenbink-Garcia, 2012).

To sum up, the emotions of students have been deliberated recurrently in the literature (Gkonou et al., 2020), while the emotions of teachers have stayed in the shades of study and theory. It is frequently supposed that the educators are the utmost significant figures on the academic prospect (Pishghadam et al., 2021a), and their role to a large degree regulates the final achievement of both scholars and the teaching organization (Mercer and Dörnyei, 2020). However, to the best of the information of a researcher, the way both learners and their teachers react to emotions should be taken into account as it seems that they may impact the teaching of students in ways that may afterward turn into their intellectual growth. Consequently, extending the information on emotions undergone in the classroom by both teachers and learners is worth investigating on the one hand and their effects on language success and achievement on the other hand.

## Emotions and Learning

Emotions can impact an individual in determining to study a language and to do activities in a classroom (Méndez Lopez and Pea Aguilar, 2013) and they added that both types of emotions, namely positive and negative, can have noteworthy effects on the enthusiasm of students. They declared that negative feelings such as anxiety and depression can boost learning, and they can also be regarded as constructive in the procedure of language education. Benesch (2017) preserved that language courses are incorporating both destructive and constructive emotions; the former hindering effective education and the latter nurturing them. Additionally, due to the interactional nature of the educator-learners relationship, they entail the incorporation of individually significant content and distinctiveness, which are simplified through relation of teachers and emotive considerations of students (Xie and Derakhshan, 2021).

The existing review of literature has acknowledged the function of emotions of teachers concerning various features of learning, namely teacher, education, learner, and scholarship. Primarily, it is extensively documented that emotion of teachers influences their teaching. The emotion of a teacher interweaves with the intellect of educators and impetus that is correlated with their teaching manners (Uitto et al., 2015). It is acknowledged that the emotion of a teacher impacts numerous facets of the perceptive procedures of teachers. For instance, they can stimulus their consideration, recall, and thoughtfulness (Golombek and Doran, 2014). In addition, other results demonstrated that the emotion of a teacher is connected to other features such as teacher identity, helplessness, individual and professional lives, and well-being (Schutz and Zembylas, 2009; Kelchtermans, 2011; Lee et al., 2013; Yin et al., 2017; Greenier et al., 2021). Certainly, emotions had a momentous effect on teacher growth in the instruction career (Fried et al., 2015). Furthermore, studies also have proved that emotions of teachers influence many parts of time of students in the class together with feelings of learners, learner-teacher relationships, engagement, stress, and enthusiasm (Becker et al., 2014; Van Uden et al., 2014; Fathi and Derakhshan, 2019; Kruk, 2021; Wang and Derakhshan, 2021).

Empirical studies specified that the rapport between educators and learners is a perilous “emotive filter” (Zembylas, 2014). Nevertheless, it is suggested that educators want proper maintenance to progress a noble learner-teacher rapport in which a range of emotive determinations is restricted (Newberry, 2010). It is believed that educators with destructive feelings are prone to diminish the probabilities that learners will employ a higher degree of intellectual learning methods (Linnenbrink-Garcia and Pekrun, 2011). Teaching spaces that determine constructive emotions are more about generating a superior educational atmosphere that preserves the success and development of learners (Yan et al., 2011; Pishghadam et al., 2021b).

### The Control-Value Theory

The control-value theory incorporates suggestions from expectancy value and attributional ways to deal with attainment emotions (Turner and Schallert, 2001) that are characterized as feelings identified with accomplishment exercises and their results of either success or disappointment. The theory sets that these feelings are stimulated by psychological evaluations of control over, and the emotional worth of, accomplishment exercises as well as their results (Pekrun and Perry, 2014). Control examinations comprise of the impression of capability of a person to effectively accomplish activities (i.e., scholarly self-ideas assumptions) and to achieve results (result assumptions). Worth examinations relate to the apparent significance of these exercises and their results (Pekrun et al., 2017). Moreover, the theory puts forward that these feelings, thus, impact accomplishment behavior and execution. Since execution results shape the ensuing impression of control over execution, one significant suggestion is that feelings, their examination precursors, and their exhibition results are connected by mutual causality. As far as mutual causality, the theory is predictable with corresponding impacts models for factors like self-ideas of learners (Marsh et al., 2005; Marsh and Craven, 2006).

### The Broaden-and-Build Theory

As stated by Fredrickson (2004), the broaden-and-build theory proposes that constructive feelings work to broaden temporary thought-action collections of people and construct their permanent subjective foundations. The theory suggests three explicit impacts of positive emotions, including the widening of thought-action collections, the assembling of assets for the future, and the undoing of bothersome impacts of negative feelings (Fredrickson, 2013). Positive emotions empower practices like play, innovativeness, interest, and investigation, which are generally seen as advantageous in learning. These expanding practices differ from those created by a destructive emotive reaction. When tension is hard to stay away from, positive emotions might even function as a precaution or defensive role contrary to negative feelings, like language nervousness (MacIntyre, 2017). Along with the broaden-and-build theory, the ramifications of bringing constructive feelings into language education could expand further than that of diminishing or fixing the impacts of negative feelings. This theory proposes that positive emotions expand the viewpoint of an individual student, working with commitment language, and investigation inside new settings. Such exercises may permit students to better observe L2 input (Mackey, 2006), assisting construct assets by including explicit language encounters coming from relational communications that gather social principle of students (Gregersen et al., 2014).

This theory presents that positive emotions work in at least four significant ways (Cohn and Fredrickson, 2006). First, to begin with, positive emotions will, in general, expand consideration and thinking of individuals, prompting an investigation and new learning. Second, positive feeling assists with fixing the lingering impacts of negative emotional encouragement. The third role of positive feeling is to advance versatility by setting off useful responses to distressing occasions, like working on cardiovascular recuperation and developing salient sensations of satisfaction and interest while under pressure. Fourth, positive feeling advances building individual assets, for example, social bonds brought about by grins, scholarly assets sharpened during innovative play, and even when younger practice self-safeguarding moves during a play.

## Implications and Future Directions

The arrival of PP in language education and the specific domain of feeling and language learning resonates with a difference in attitude among language specialists, moving from a destructive concentration to a more positive, adjusted methodology for examining language education. PP is considered to be an interesting theme to bring into the investigation of feelings and language learning since it incorporates aspects like positive foundations, attributes, and emotions, all of which are among essential pertinence to the assessment and development of advantageous effective encounters in language classes. The review introduced in this study stresses the requirement for the investigation of feelings to have a more focal situation in principle, exploration, and practice in language learning. While the comprehension and administration of feelings are significant components of information and capacity of an educator, for students, feelings are significant in terms of how they explore and process their education. The present paper can familiarize language scholars, experts, teachers, and students with the main values of emotions and their use in language education.

The present review has significant implications for language instruction as well future inquiries in second language acquisition (SLA) research. The organization of emotions is a central element of awareness and capability of a teacher, while for students, they are fundamental to how they direct and develop their education. In the language setting, developing positive emotions and reducing negative feelings of both educators and students could enhance inspiration, engagement, and achievement.

Learners inclined to positive emotions might encounter undeniable degrees of accomplishment mostly in light of the fact that such feelings are related to high-quality connections with friends and educators. Presently, the emotion of an educator is considered as a crucial research focus for several reasons that assume a critical and essential part in language learning and the well-being of learners (Fried et al., 2015). At present, educators are established to practice more negative than positive emotions (Thompson, 2014; Hassard et al., 2016) and educators have hurt from the same problematic and challenging issues that have been even worse recently (Yang et al., 2019). The emotion of a teacher has been acknowledged to affect the passionate situation in a classroom; thanks to its effects on learner-educator communications and teaching space manners (Schroeder, 2006). This burden has not facilitated the prevailing emotive state of educators (Thompson, 2014). In the same vein, the emotions of teachers affect their individual and proficient lives and eventually govern teacher efficiency (Schutz and Zembylas, 2009). Similarly, it can be asserted that the nature of education learners receive is likewise impacted by the feelings of their educators. Scholastic quality is worse among educators who show negative feelings and disappointment in instructing as opposed to those who exhibit more constructive emotions (Frenzel et al., 2011; Postareff and Lindblom-Ylänne, 2011). First, regardless of whether positive or negative, the feelings of educators have a specific impact on their enthusiasm (Sutton and Wheatley, 2003). Positive emotions are a significant, but not an entirely enveloping, component of intrinsic motivation. Notwithstanding, the intrinsic motivational level of the educator is often lowered by negative emotions (Sutton and Wheatley, 2003). Second, the teaching approach is closely connected to the accomplishment of education of learners, which is firmly identified with the feelings of the educator (Mohammadjani and Tonkaboni, 2015).

The underlying empirical proof created in this review paper is delivering new thoughts, information, and experiences for L2 emotion researchers (Lee, 2014) and suggesting useful proposals for educators (Shao et al., 2019). The new advancement of investigations on emotions in psychology and teaching gives L2 teachers invigorating potential outcomes. Concerning language educators, they are prescribed to acquire information on psychology and get to know the significant ideas and segments of feelings of educators and to attempt to show their insight in their practice and states of mind and to invigorate the improvement of perspectives of students on the accomplishment of educators in the language classes. Furthermore, by teaching to determine personal aims, educators can motivate learners. They can urge students to attain self-satisfaction in completing an assignment effectively. The educators can practice making light of the negative emotions, since it is perilous to concentrate a lot on negative emotions because showing such impeding factors in the classes will have a bad effect on some learners. The incorporation of emotion paradigms into the language learning field not only helps to control current shortfalls in language inquiries, for example, procedural matters and assessment difficulties, but also to provide new chances for the research populations of learning and psychology.

Regarding the language learners, they are recommended to concentrate on their emotions of educators and captivate the types of emotions, namely, pleasure in the educational field and a sensation of pleasure, and do not allow the destructive emotions to impact their fulfillment of learning a language and comprehend the ways wherein they are pursuing to grasp their scholastic proficiencies and for teachers especially to disclose the method in which students follow their language learning capabilities in the educational setting.

Syllabus designers should think through employing teaching techniques and strategies and utilizing mediation tasks and activities that nurture opinions of control of learners on their learning package to uphold their success both through increasing views of control, and by enlightening positive feelings and decreasing negative ones (Hamm et al., 2017). They are supposed to plan tasks based on desires, emotional states, and well-being of students that focus on the roles of emotions of educators and how the learners deduce their feelings, and the special effects of opinions of learners in the educational process. Since it is believed that achievement from emotion investigation in psychology and learning knowledge can support fundamental awareness into language achievement. It may function as a new stimulus in language education to plan further research for inspecting the emotive features of success.

Finally, future scholars can conduct some research incorporating the views of diverse participants in this respect; therefore, enthusiastic researchers are suggested to study these constructs regarding other factors such as optimism, pleasure, engagement, and social behaviors such as intelligibility, credibility, and immediacy (Derakhshan, 2021). In particular, some empirical studies embedded in the theoretical rational aforementioned can be done to add the fresh and nutritious food to the emotional studies of SLA. As has been indicated, emotional factors in language education have not been equally investigated (Wang et al., 2021). In view of this, it is advocated that EFL teachers of the globe and applied linguists should put their effort into doing the related studies to flourish the emotional studies. Aside from this, a large body of research is quantitative rather than qualitative studies. The main reason for this may be due to the fact that it is less challenging for language educators to collect their research data. As is known, it is pretty time-consuming to collect and codify the interview protocols as well as reflective journals. Therefore, it is suggested that more qualitative studies be conducted in the near future. Last but not least, cross-cultural or cross-national studies should also be done because the variables of emotion-based education will, to some great extent, be influenced by the identity of participants.

## Author Contributions

QH: conceptualization, writing of the draft, and checking language before this manuscript was ready for submission.

## Conflict of Interest

The author declares that the research was conducted in the absence of any commercial or financial relationships that could be construed as a potential conflict of interest.

## Publisher’s Note

All claims expressed in this article are solely those of the authors and do not necessarily represent those of their affiliated organizations, or those of the publisher, the editors and the reviewers. Any product that may be evaluated in this article, or claim that may be made by its manufacturer, is not guaranteed or endorsed by the publisher.

## References

[B1] Agudo J. D. D. M. (2018). *Emotions in second language teaching: Theory, research and teacher education.* New York: Springer, 10.1007/978-3-319-75438-3-20

[B2] Becker E. S. Goetz T. Morger V. Ranellucci J. (2014). The importance of teachers’ emotions and instructional behavior for their students’ emotions: an experience sampling analysis. *Teach. Teach. Educ.* 43 15–26. 10.1016/j.tate2014.05.002

[B3] Beilock S. L. Gunderson E. A. Ramirez G. Levine S. C. (2010). Female teachers’ math anxiety affects girls’ math achievement. *Proc. Natl. Acad. Sci. U. S. A.* 107 1060–1063. 10.1073/pnas.0910967107 20133834 PMC2836676

[B4] Benesch S. (2012). *Considering Emotions In Critical English Language Teaching: Theories And Practice.* NewYork: Routledge.

[B5] Benesch S. (2017). *Emotions And English Language Teaching: Exploring Teachers’ Emotion Labor.* New York: Routledge.

[B6] Bigelow M. (2019). (Re)considering the role of emotion in language teaching and learning. *Mod. Lang. J.* 103 515–516. 10.1111/modl.12569

[B7] Chang M. L. (2009). An appraisal perspective of teacher burnout: examining the emotional work of teachers. *Educ. Psychol. Rev.* 21 193–218. 10.1007/s10648-009-9106-y

[B8] Cohn M. A. Fredrickson B. L. (2006). Beyond the moment, beyond the self: shared ground between selective investment theory and the broaden-and-build theory of positive emotions. *Psychol. Inq.* 17 39–44.

[B9] Daubney M. Dewaele J. M. Gkonou C. (2017). “Introduction: Preliminary thoughts on language anxiety and the focus of this anthology,” in *New Insights Into Language Anxiety: Theory, Research And Educational Implications*, eds Gkonou C. Daubney M. Dewaele J. M. (Blue RidgeSummit: Multilingual Matters), 1–7.

[B10] Day C. Qing G. (2009). “Teacher emotions: Well-being and effectiveness,” in *Advances in teacher emotion research: The impact on teachers’ lives*, eds Schutz P. A. Zembylas M. (New York: Springer), 15–32.

[B11] Derakhshan A. (2021). The predictability of Turkman students’ academic engagement through Persian language teachers’ nonverbal immediacy and credibility. *J. Teach. Persian Speak. Lang.* 10 3–26. 10.30479/JTPSOL.2021.14654.1506

[B12] Derakhshan A. Coombe C. Arabmofrad A. Taghizadeh M. (2020). Investigating the effects of English language teachers’ professional identity and autonomy in their success. *Issues Lang. Teach.* 9 1–28. 10.22054/ilt2020.52263.496

[B13] Derakhshan A. Kruk M. Mehdizadeh M. Pawlak M. (2021). Boredom in online classes in the Iranian EFL context: sources and solutions. *System* 101:102556. 10.1016/jsystem.2021.102556

[B14] Derakhshan A. Saeidi M. Beheshti F. (2019). The interplay between Iranian EFL teachers’ conceptions of intelligence, care, feedback, and students’ stroke. *IUP J. English Stud.* 14 81–98.

[B15] Dewaele J. M. (2005). Investigating the psychological and emotional dimensions in instructed language learning: obstacles and possibilities. *Mod. Lang. J.* 89 367–380.

[B16] Dewaele J. M. (2015). On emotions in foreign language learning and use. *Lang. Teach.* 39 13–15. 10.37546/JALTTLT39.3-3

[B17] Dewaele J. M. (2018). Why the dichotomy ‘L1 versus LX user’ is better than ‘native versus non-native speaker. *App. Linguist.* 39 236–240. 10.1093/applin/amw055

[B18] Dewaele J. M. Alfawzan M. (2018). Does the effect of enjoyment outweigh that of anxiety in foreign language performance? *Stud. Second Lang. Learn. Teach.* 8 21–45. 10.14746/ssllt.2018.8.1.2

[B19] Dewaele J. M. Chen X. Padilla A. M. Lake J. (2019). The flowering of positive psychology in foreign language teaching and acquisition research. *Front. Psychol.* 10 21–28. 10.3389/fpsyg20:19.02128 31607981 PMC6769100

[B20] Dewaele J. M. Li C. (2020). Emotions in second language acquisition: a critical review and research agenda. *Foreign Lang. World* 1 34–49.

[B21] Dewaele J. M. MacIntyre P. (2014). The two faces of Janus? Anxiety and enjoyment in the foreign language classroom. *Stud. Second Lang. Learn. Teach.* 4 237–274. 10.14746/ssllt.2014.4.2.5

[B22] Dewaele J. M. Witney J. Saito K. K. Dewaele L. (2017). Foreign language enjoyment and anxiety: the effect of teacher and learner variables. *Lang. Teach. Res.* 22 676–697. 10.1177/1362168817692161

[B23] Dörnyei Z. (2005). *The Psychology Of The Language Learner: Individual Differences In Second Language Acquisition.* New Jersey: Lawrence Erlbaum Associates Publishers.

[B24] Ergün A. L. P. Dewaele J. M. (2021). Do well-being and resilience predict the foreign language teaching enjoyment of teachers of Italian?. *System* 99 102–506. 10.1016/j.system.2021.102506

[B25] Fathi J. Derakhshan A. (2019). Teacher self-efficacy and emotional regulation as predictors of teaching stress: an investigation of Iranian English language teachers. *Teach. English Lang.* 13 117–143.

[B26] Fathi J. Greenier V. Derakhshan A. (2021). Teacher self-efficacy, reflection, and burnout among Iranian EFL teachers: the mediating role of emotion regulation. *Iran. J. Lang. Teach. Res.* 9 13–37.

[B27] Fredrickson B. Losada M. (2005). Positive affect and the complex dynamics of human flourishing. *Am. Psychol.* 60 678–686. 10.1037/0003-066X.60.7.678 16221001 PMC3126111

[B28] Fredrickson B. L. (2003). The value of positive emotions: the emerging science of positive psychology is coming to understand why it’s good to feel good. *Am. Sci.* 91 330–335. 10.1511/2003.4.330

[B29] Fredrickson B. L. (2004). The broaden-and-build theory of positive emotions. *Philos. Trans. Biol. Sci.* 359 1367–1377. 10.1098/rstb.2004.1512 15347528 PMC1693418

[B30] Fredrickson B. L. (2013). “Positive emotions broaden and build,” in *Advances In Experimental Social Psychology*, eds Devine P. Plant A. (Burlington: Academic Press), 1–53.

[B31] Frenzel A. C. Götz T. Stephens E. J. Jacob B. (2009). “Antecedents and effects of teachers’ emotional experiences: An integrative perspective and empirical test,” in *Advances In Teacher Emotions Research: The Impact On Teachers Lives*, eds Schutz P. A. Zembylas M. (New York: Springer), 129–148.

[B32] Frenzel A. C. Götz T. Stephens E. J. Jacob B. (2011). Antecedents and effects of teachers’ emotional experiences: an integrated perspective and empirical test. 129–151.

[B33] Fried L. Mansfield C. Dobozy E. (2015). Teacher emotion research: introducing a conceptual model to guide future research. *Issues Educ. Res.* 25 415–441.

[B34] Ge Z. G. Zhang A. Y. Li Y. F. Su J. (2019). Exploring the impact of teachers’ verbal immediacy as an emotion mediating factor on adult e-learners’ language learning. *J. Educ. Technol. Soc.* 22 77–89.

[B35] Gkonou C. Dewaele J. M. King J. (2020). “Introduction to the emotional rollercoaster of language teaching,” in *The Emotional Rollercoaster Of Language Teaching*, eds Gkonou C. Dewaele J. M. King J. (Bristol: Multilingual Matters), 1–12.

[B36] Goetz T. Frenzel A. C. Stoeger H. Hall N. C. (2010). Antecedents of everyday positive emotions: an experience sampling analysis. *Motiv. Emot.* 34 49–62.

[B37] Golombek P. Doran M. (2014). Unifying cognition, emotion, and activity in language teacher professional development. *Teach. Teach. Educ.* 39 102–111. 10.1016/j.tate.2014.01.002

[B38] Greenier V. Derakhshan A. Fathi J. (2021). Emotion regulation and psychological well-being in teacher work engagement: a case of British and Iranian English language teachers. *System* 97:102446. 10.1016/j.system.2020.102446

[B39] Gregersen T. Macintyre P. D. Meza M. D. (2014). The motion of emotion: idiodynamic case studies of learners’ foreign language anxiety. *Mod. Lang. J.* 98 574–588. 10.1111/j.1540-4781.2014.12084.x

[B40] Hagenauer G. Volet S. E. (2014). I don’t hide my feelings, even though I try to: insight into teacher educator emotion display. *Aust. Educ. Res.* 41 261–281. 10.1007/s13384-013-0129-5 26472919 PMC4598951

[B41] Hamm J. M. Perry R. P. Chipperfield J. G. Murayama K. Weiner B. (2017). Attribution-based motivation treatment efficacy in an online learning environment for students who differ in cognitive elaboration. *Motiv. Emot.* 41 600–616. 10.1007/s11031-017-9632-8

[B42] Hassard J. Teoh K. Cox T. (2016). Organizational uncertainty and stress among teachers in Hong Kong: work characteristics and organizational justice. *Health Promot. Int.* 32 860–870. 10.1093/heapro/daw018 27030559

[B43] Hosotani R. Imai-Matsumura K. (2011). Emotional experience, expression, and regulation of high-quality Japanese elementary school teachers. *Teach. Teach. Educ.* 27 1039–1048. 10.1016/j.tate.2011.03.010

[B44] Ismail N. M. (2015). EFL Saudi students’ class emotions and their contributions to their English achievement at Taif University. *Int. J. Psychol. Stud.* 7 19–42. 10.5539/ijps.v7n4p19

[B45] Kelchtermans G. (2011). “Vulnerability in teaching: The moral and political roots of structural conditions,” in *New understanding of teachers’ work: Emotions and educational change*, eds Day C. Lee J. C. K. (New York: Springer), 65–82. 10.1080/03054985.2010.545192

[B46] Keller M. M. Chang M. L. Becker E. S. Goetz T. Frenzel A. C. (2014). Teachers’ emotional experiences and exhaustion as predictors of emotional labor in the classroom: an experience sampling study. *Front. Psychol.* 5:1442. 10.3389/fpsyg.2014.01442 25566124 PMC4263074

[B47] King R. B. Chen J. (2019). Emotions in education: asian insights on the role of emotions in learning and teaching. *Asia Pac. Educ. Res* 28 279–281. 10.1007/s40299-019-00469-x

[B48] Kruk M. (2021). *Investigating Dynamic Relationships Among Individual Difference Variables In Learning English As A Foreign Language In A Virtual World.* Switzerland: Springer.

[B49] Lee J. C. K. Huang Y. X. H. Law E. H. F. Wang M. H. (2013). Professional identities and emotions of teachers in the context of curriculum reform: a Chinese perspective. *Asia Pac. J. Teach. Educ.* 41 271–287. 10.1080/1359866X.2013.809052

[B50] Lee M. (2014). *Achievement goals, emotions, and foreign language performance in German and Korean students*. Ph. D. thesis, University of Munich, Munich.

[B51] Li C. (2020). A Positive Psychology perspective on Chinese EFL students’ trait emotional intelligence, foreign language enjoyment and EFL learning achievement. *J. Multiling. Multicult. Dev.* 41 246–263. 10.1080/01434632

[B52] Li C. Dewaele J.-M. Jiang G. (2019). The complex relationship between classroom emotions and EFL achievement in China. *Appl. Linguist. Rev.* 11 485–510. 10.1515/applirev-2018-0043

[B53] Linnenbrink-Garcia L. Pekrun R. (2011). Students’ emotions and academic engagement: introduction to the special issue. *Contemp. Educ. Psychol.* 36 1–3. 10.1016/j.cedpsych.2010.11.004

[B54] MacIntyre P. D. (2002). “Motivation, anxiety, and emotion in second language acquisition,” in *Individual Differences And Instructed Language Learning*, ed. Robinson P. (Amsterdam: John Benjamin), 45–68.

[B55] MacIntyre P. D. (2017). “An overview of language anxiety research and trends in its development,” in *New insights into language anxiety: Theory, research and educational implications*, eds Gkonou C. Daubney M. Dewaele J. M. (Bristol: Multilingual Matters), 25–53.

[B56] MacIntyre P. D. Gregersen T. (2012). Emotions that facilitate language learning: the positive-broadening power of the imagination. *Stud. Second Lang. Learn.Teach.* 2 193–213. 10.14746/ssllt.2012.2.2.4

[B57] MacIntyre P. D. Gregersen T. Mercer S. (2016). *Positive psychology in SLA.* Bristol: Multilingual Matters.

[B58] MacIntyre P. D. Mercer S. (2014). Introducing positive psychology to SLA. *Stud. Second Lang. Learn. Teach.* 4 153–172. 10.14746/ssllt.2014.4.2.2

[B59] Mackey A. (2006). Feedback, noticing and instructed second language learning. *Appl. Linguist.* 27 405–430. 10.1093/applin/ami051

[B60] Marsh H. W. Craven R. G. (2006). Reciprocal effects of self-concept and performance from a multidimensional perspective. *Perspect. Psychol. Sci.* 1 133–163. 10.1111/j.1745-6916.2006.00010.x 26151468

[B61] Marsh H. W. Trautwein U. Lüdtke O. Köller O. Baumert J. (2005). Academic self-concept, interest, grades, and standardized test scores: reciprocal effects models of causal ordering. *Child Dev.* 76 397–416. 10.1111/j.1467-8624.200500853.x 15784090

[B62] Mendez Lopez M. G. Pea Aguilar A. P. (2013). Emotions as learning enhancers of foreign language learning motivation. *Profile* 15 109–124.

[B63] Méndez Lopez M. G. Pea Aguilar A. P. (2013). Emotions as learning enhancers of foreign language learning motivation. *Profile* 15 109–124.

[B64] Mercer S. Dörnyei Z. (2020). *Engaging Language Learners In Contemporary Classrooms.* Cambridge: Cambridge University Press.

[B65] Mercer S. Kostoulas A. (2018). “Introduction to language teacher psychology,” in *Language Teacher Psychology*, eds Mercer S. Kostoulas A. (Bristol: Multilingual Matters), 1–17.

[B66] Mohammadjani F. Tonkaboni F. (2015). A comparison between the effect of cooperative Learning teaching method and lecture teaching method on students’ learning and satisfaction level. *Int. Educ. Stud.* 8 107–112. 10.5539/ies.v8n9p107

[B67] Newberry M. (2010). Identified phases in the building and maintaining of positive teacher–student relationships. *Teach. Teach. Educ.* 26 1695–1703. 10.1016/j.tate.2010.06.022

[B68] Pawlak M. Zawodniak J. Kruk M. (2020). *Boredom In The Foreign Language Classroom: A Micro-Perspective.* Switzerland: Springer Nature.

[B69] Pekrun R. Goetz T. Titz W. Perry R. P. (2002). Academic emotions in students’ self-regulated learning and achievement: a program of qualitative and quantitative research. *Educ. Psychol.* 37 91–105.

[B70] Pekrun R. Lichtenfeld S. Marsh H. W. Murayama K. Goetz T. (2017). Achievement emotions and academic performance: longitudinal models of reciprocal effects. *Child Dev.* 88 1653–1670. 10.1111/cdev.12704 28176309

[B71] Pekrun R. Linnenbink-Garcia L. (2012). “Academic emotions and student engagement,” in *Handbook Of Research On Student Engagement*, eds Christenson S. L. Reschly A. L. Wylie C. (New York: Springer), 259–282.

[B72] Pekrun R. Murayama K. Marsh H. W. Goetz T. Frenzel A. C. (2019). Happy fish in little ponds: testing a reference group model of achievement and emotion. *J. Pers. Soc. Psychol.* 117 166–185. 10.1037/pspp0000230 30667258

[B73] Pekrun R. Perry R. P. (2014). “Control-value theory of achievement emotions,” in *International Handbook Of Emotions In Education*, eds Pekrun R. Linnenbrink-Garcia L. (New York: Taylor & Francis), 120–141.

[B74] Pishghadam R. Derakhshan A. Jajarmi H. Tabatabaee Farani S. Shayesteh S. (2021a). Examining the role of teachers’ stroking behaviors in EFL learners’ active/passive motivation and teacher success. *Front. Psychol.* 12:707314. 10.3389/fpsyg.2021.707314 34354648 PMC8329251

[B75] Pishghadam R. Derakhshan A. Zhaleh K. Habeb Al-Obaydi L. (2021b). Students’ willingness to attend EFL classes with respect to teachers’ credibility, stroke, and success: a cross-cultural study of Iranian and Iraqi students’ perceptions. *Curr. Psychol.* 40 1–15. 10.1007/s12144-021-01738-z

[B76] Postareff L. Lindblom-Ylänne S. (2011). Emotions and confidence within teaching in higher education. *Stud. High. Educ.* 36 799–813. 10.1080/03075079.2010.483279

[B77] Richards J. C. (2020). Exploring emotions in language teaching. *RELC J.* 1–15. 10.1177/0033688220927531

[B78] Ross M. L. (2015). What have we learned about the resource curse?. *Annu. Rev. Polit. Sci.* 18 239–259.

[B79] Saito K. Dewaele J. Abe M. In’nami Y. (2018). Motivation, emotion, learning experience, and second language comprehensibility development in classroom settings: a cross-sectional and longitudinal study. *Lang. Learn.* 68 709–743. 10.1111/lang.12297

[B80] Schroeder L. (2006). *What High-Stakes Testing Means For The Emotional Well-Being Of Students And Teachers*. Ph.D. thesis, Claremont Graduate University, Claremon.

[B81] Schutz P. A. Zembylas M. (2009). *Advances in Teacher emotion Research: The impact on teachers’ lives.* New York: Springer.

[B82] Seli P. Wammes J. D. Risko E. F. Smilek D. (2016). On the relation between motivation and retention in educational contexts: the role of intentional and unintentional mind wandering. *Psychon. Bull. Rev.* 23 1280–1287. 10.3758/s13423-015-0979-0 26585116

[B83] Shao K. Pekrun R. Nicholson L. (2019). Emotions in classroom language learning: what can we learn from achievement emotion research?. *System* 86 102–121. 10.1016/j.system.2019.102121

[B84] Sharwood Smith M. (2017). *Introducing Language and Cognition. A Map of the Mind.* Cambridge: Cambridge University Press.

[B85] Sutton R. E. Harper E. (2009). “Teachers’ emotion regulation,” in *International Handbook Of Research On Teachers And Teaching*, eds Saha L. J. Dworkin A. G. (New York: Springer), 389–401.

[B86] Sutton R. E. Wheatley K. F. (2003). Teachers’ emotions and teaching: a review of the literature and directions for future research. *Educ. Psychol. Rev.* 15 327–358.

[B87] Swain M. (2013). The inseparability of cognition and emotion in second language learning. *Lang. Teach.* 46 195–207. 10.1016/j.dental.2013.01.002 23410552

[B88] Taxer J. L. Frenzel A. C. (2015). Facets of teachers’ emotional lives: a quantitative investigation of teachers’ genuine, faked, and hidden emotions. *Teach. Teach. Educ.* 49 78–88. 10.1016/j.tate.2015.03.003

[B89] Thompson J. N. (2014). *Interaction and Coevolution.* Chicago: University of Chicago Press, 10.7208/9780226127323

[B90] Turner J. E. Schallert D. L. (2001). Expectancy value relationships of shame reactions and shame resiliency. *J. Educ. Psychol.* 93 320–329. 10.1037/0022-0663.93.2.320

[B91] Tyng C. M. Amin H. U. Saad M. N. M. Malik A. S. (2017). The influences of emotion on learning and memory. *Front. Psychol.* 8:1454. 10.3389/fpsyg.2017.01454 28883804 PMC5573739

[B92] Uitto M. Jokikokko K. Estola E. (2015). Virtual special issue on teachers and emotions in Teaching and Teacher Education (TATE) in 1985-2014. *Teach. Teach. Educ.* 50 124–135. 10.1016/j.tate.2015.05.008

[B93] Van Uden J. M. Ritzen H. Pieters J. M. (2014). Engaging students: the role of teacher beliefs and interpersonal teacher behavior in fostering student engagement in vocational education. *Teach. Teach. Educ.* 37 21–32. 10.1016/J.TATE.2013.08.005

[B94] Wang Y. L. Derakhshan A. (2021). [Review of the book Investigating dynamic relationships among individual difference variables in learning English as a foreign language in a virtual world, by M. Kruk]. *System* 100 102531. 10.1016/j.system.2021.102531

[B95] Wang Y. L. Derakhshan A. Zhang L. J. (2021). Researching and Practicing Positive Psychology in Second/Foreign Language Learning and Teaching: the Past, Current and Future Directions. *Front. Psychol.* 12:731721. 10.3389/fpsyg.2021.731721 34489835 PMC8417049

[B96] White C. J. (2018). “The Emotional Turn in Applied Linguistics and TESOL: Significance, Challenges and Prospects,” in *Emotions in Second Language Teaching*, ed. de Dios Martínez Agudo J. (Berlin: Springer), 125–141.

[B97] Xie F. Derakhshan A. (2021). A conceptual review of positive teacher interpersonal communication behaviors in the instructional context. *Front. Psychol.* 12:708490. 10.3389/fpsyg.2021.708490 34335424 PMC8319622

[B98] Yan E. M. Evans I. M. Harvey S. T. (2011). Observing emotional interactions between teachers and students in elementary school classrooms. *J. Res. Child. Educ.* 25 82–97. 10.1080/02568543.2011.533115

[B99] Yang R. You X. Zhang Y. Lian L. Feng W. (2019). Teachers’ mental health becoming worse: the case of China. *Int. J. Educ. Dev.* 70:102077. 10.1016/j.ijedudev.2019.102077

[B100] Yin H. B. Huang S. Wang W. (2017). Work environment characteristics and teacher well-being: the mediation of emotion regulation strategies. *Int. J. Environ. Res. Public Health* 13 907–923. 10.3389/fchem.2017.00123 27649216 PMC5036740

[B101] Zawodniak J. Kruk M. Pawlak M. (2021). Boredom as an aversive emotion experienced by English majors. *RELC J.* 10.1177/0033688220973732

[B102] Zembylas M. (2014). The place of emotion in teacher reflection: elias, Foucault and ‘critical emotional reflexivity’. *Power Educ.* 6 210–222. 10.2304/power2014.6.2.210 18307325

[B103] Zhang L. Tsung L. (2021). Learning Chinese as a second language in China: positive emotions and enjoyment. *System* 96 102–410. 10.1016/j.system.2020.102410

